# A Study on the Durability of Solidification Materials Based on Multi-Source Solid Waste Using Recycled Aggregates in Chemical Environments

**DOI:** 10.3390/ma19183949

**Published:** 2026-09-17

**Authors:** Jiaojiao Ni, Yongqi Zhao, Qing Jiang, Haitao Hu, Haowei Ding, Qiwei Zhan

**Affiliations:** 1 China Construction Industrial & Energy Engineering Group Co., Ltd., Nanjing 210046, China; 2 School of Civil Engineering and Architecture, Jiangsu University of Science and Technology, Zhenjiang 212100, China; 3 School of Transportation, Southeast University, Nanjing 211189, China

**Keywords:** mixed-source solid waste, recycled concrete aggregate, chemical erosion, durability, microstructure

## Abstract

Against the backdrop of global green and low-carbon development, recycling industrial solid waste for building-material applications attracts increasing attention. In this work, a composite solidifier (SGPC) was prepared using soda residue (SR), ground-granulated blast-furnace slag (GGBS), phosphogypsum (PG) and Portland cement. Fluidized solidified soil was produced by incorporating 15% recycled concrete aggregate (RCA). Chemical-erosion tests including strong-acid, strong-alkaline and neutral-sulfate corrosion were carried out. Mass-loss-rate and unconfined-compressive-strength-loss-rate measurements at different exposure ages, combined with X-ray diffraction (XRD) and scanning electron microscopy (SEM) characterisation, were adopted to investigate the macroscopic durability, hydration-product phases and microstructural features of RCA-modified solidified soil. The test results show that under acid corrosion, the 120 d strength-loss rate decreases from 19.86% (RCA-free group) to 9.02% for specimens containing 15% RCA. Under alkaline corrosion, the 120-d mass-loss rate of the RCA-modified group reaches only 0.33%, much lower than 1.46% of the group without RCA. Under sulfate corrosion, the 120-d strength-loss rate drops from 5.65% to 2.02% after RCA addition. This work provides experimental data and theoretical support for the joint utilisation of multi-source solid waste and recycled aggregates in soil-solidification engineering.

## 1. Introduction

The co-processing of bulk industrial solid waste and construction and demolition waste to produce cementitious solidification materials has become a key direction for green and low-carbon development in the geotechnical engineering field [1]. Industrial by-products such as granulated blast furnace slag (GGBS), phosphogypsum (PG), and soda ash residue (SR) are stockpiled in massive quantities, not only occupying land resources but also posing environmental risks such as soil and water pollution. At the same time, the process of urban renewal generates large amounts of waste concrete; however, the resource utilization rate of recycled concrete aggregate (RCA)—obtained by crushing this waste—remains low due to issues such as high porosity, high water absorption, and poor interfacial properties [2,3,4,5]. The collaborative development of novel cementitious solidification materials using various types of solid waste—to replace traditional silicate cement systems with high carbon emissions—for engineering applications such as the reinforcement of soft soils, site remediation, and roadbed backfilling has gradually become the mainstream direction of technological development within the industry [6,7,8]. GGBS from steelmaking, SR from the chemical industry, and PG—a byproduct of phosphate fertilizer production—are representative bulk industrial solid wastes, with enormous annual generation. Long-term open-air stockpiling not only occupies vast land areas, wasting land resources, but also allows soluble salts, alkaline components, and harmful impurities present in the waste to leach into the soil or be transported by surface runoff, thereby triggering ecological and environmental issues such as soil salinization and groundwater contamination, which pose a potential threat to regional ecological security [9,10]. Meanwhile, ongoing urban renewal, old-city redevelopment, and infrastructure expansion and renovation projects are demolishing a large number of old buildings and structures, generating substantial amounts of waste concrete debris. Although waste concrete can be processed by crushing and screening to RCA, the resulting RCA suffers from inherent drawbacks, including high porosity, high water absorption, residual old mortar on the surface, and weak interfacial bonding between the aggregate and cement paste. These drawbacks limit its applications and lead to a low resource recovery rate; consequently, a large volume of construction and demolition waste is still simply landfilled, failing to realize its full resource potential.

In actual engineering applications, solidified soil—such as that used in road embankments, excavation backfill, and site remediation—is often exposed to complex chemical environments. Groundwater, industrial leachate, and saline–alkali soils and groundwater can cause multiple forms of chemical erosion, including acidic, alkaline, and sulfate corrosion [11]. Once chemical media penetrate the solidified soil, they undergo physicochemical reactions (e.g., dissolution, decomposition, and expansion) with the hydration products of the cementitious matrix, thereby damaging the internal cementitious framework and pore structure. This leads to degradation of material quality and deterioration of mechanical properties, posing a serious threat to the long-term service safety of engineering structures. Therefore, the durability of solidified materials under chemical erosion is a core indicator for evaluating the engineering applicability of solid waste-based solidification systems [12,13,14]. Currently, researchers worldwide have conducted extensive studies on the mechanical properties and durability of single solid waste-based solidification agents and RCA [15]. Wu et al. [16] used red mud and circulating fluidized bed fly ash to modify self-compacting concrete containing recycled coarse aggregate, exploiting their synergistic effects. They investigated the workability, mechanical properties, and durability of the concrete, analyzed hydration phases and microstructure, revealed the synergistic cementitious mechanism of the two solid wastes, and addressed defects inherent in recycled concrete. Wang et al. [17] employed slag-based industrial waste for solidifying silty soft soil, examining the influence of recycled fine aggregate replacement ratio on the mechanical properties of the solidified soil. By combining XRD, SEM, and thermogravimetric analysis to assess phase evolution, they elucidated the mechanism by which recycled fine aggregates regulate the microstructure of the solidification system. Teng et al. [18] incorporated a composite cementitious material consisting of soil-cement rock and solid waste into recycled aggregate concrete, conducted compressive strength tests and multiple freeze–thaw durability cycles, and used SEM to observe the evolution of microporosity and interfacial morphology in the matrix, thereby revealing the intrinsic mechanism by which the soil-cement rock content governs the mechanical and freeze–thaw durability performance of recycled concrete. However, several research gaps remain. On the one hand, most studies focus on material property evolution under a single erosion environment, with comparatively little research on multi-type chemical attacks (e.g., acid, alkaline, and saline). On the other hand, a systematic understanding of the degradation mechanisms and microscopic evolution of chemical resistance in RCA-incorporated flow-solidified soil (based on multi-source solid waste) is still lacking. The old mortar adhering to the surface of recycled aggregates exhibits potential cementitious activity; however, its synergistic interaction with the multi-source solid waste composite solidification system, along with the evolution of interfacial characteristics and phase stability in different chemical media, requires further in-depth investigation. The synergistic use of multi-source solid waste to produce low-carbon cementitious materials has become a hot research topic in the field of civil engineering. However, existing studies have primarily focused on material preparation and mechanical properties, and there is a lack of systematic understanding regarding the long-term durability evolution of these materials under complex chemical erosion environments [19]. In this study, by incorporating recycled aggregates into a solidification system using multi-source solid waste, we systematically investigated their durability performance under three typical chemical erosion conditions—acid, alkali, and salt sulfate—thereby providing experimental evidence to support the engineering application of composite solidification systems combining recycled aggregates and multi-source solid waste.

The innovative aspects of this paper are primarily reflected in the following three areas: (1) The incorporation of recycled concrete aggregate (RCA) into a composite solidification system comprising multiple industrial solid wastes (SR, GGBS, PG), and a systematic investigation of the modification and synergistic effects of RCA under three typical chemical erosion environments—acidic, alkaline and sulphate. (2) Overcoming the limitations of studies focusing on a single corrosive medium, this study conducts a parallel comparison of three chemical conditions—strong acid (pH = 2), strong alkali (pH = 13) and neutral sulphate (pH = 7)—to clarify the differences in durability of this solidification system under various media. (3) Through multi-scale combined characterisation—ranging from macroscopic properties (strength/mass loss) and phase evolution (XRD) to microscopic morphology (SEM) and quantitative analysis of hydration products (TG-DTG)—this study reveals the triple synergistic anti-erosion mechanism of RCA, namely “pore regulation, skeletal support, and induction of secondary hydration”, thereby providing a theoretical basis for the durability design of solid waste-based solidified materials.

This paper presents the preparation of a multi-source solid waste composite solidifier using a blend of SR, GGBS, PG, and Portland cement, along with the incorporation of RCA into fluidized solidified soil. Three typical chemical erosion tests—strong acid, strong alkali, and neutral sulfate—were systematically conducted. By measuring the mass loss and unconfined compressive strength (UCS) loss rates of specimens at various exposure durations, and by combining these measurements with X-ray diffraction (XRD) and scanning electron microscopy (SEM) analyses, this study reveals the effects of RCA on the macroscopic durability, hydration product phase composition, and microstructure of solidified soil under chemical attack, and elucidates both the degradation mechanisms in different corrosive media and the synergistic enhancement mechanism conferred by aggregate modification. The findings provide experimental evidence and theoretical support for the synergistic utilization of multi-source solid waste and recycled aggregates in soil solidification projects. They also serve as a reference for optimizing mix designs, guiding engineering applications, and evaluating the long-term performance of green solidification materials in complex chemical environments, thereby promoting the efficient resource utilization of construction and industrial solid waste in geotechnical engineering.

## 2. Materials and Methods

### 2.1. Materials

The soil used in this experiment was natural undisturbed soil collected from Dantu District, Zhenjiang City. After screening to remove coarse impurities and grinding in a ball mill, the soil was passed through a 16-mesh standard sieve (1 mm) to ensure a uniform particle size distribution. Its physical properties are presented in Table 1. RCA was obtained by crushing and screening waste concrete; it has a particle size range of 0.15–2 mm with continuous grading, and the particle surfaces are coated with some old mortar [20]. The RCA was purchased from Xiamen Xing Tong Building Materials Co., Ltd. (Xiamen, Fujian, China). The SGPC used in the tests consists of SR, GGBS, PG, and OPC. SR provides a stable alkaline environment; GGBS undergoes hydration under alkaline conditions to form cementitious products; PG supplies SO_4_^2−^ and a Ca^2+^ to promote hydration product formation; and OPC ensures early strength. The chemical composition of the curing agent is shown in Table 2. Analytical-grade hydrochloric acid (supplied by Sinopharm Chemical Reagents Co., Ltd., Shanghai, China) was prepared as an aqueous solution at pH 2 to simulate the corrosive effect of an acidic environment on fluidized solidified soil, thereby evaluating the material’s durability under strongly acidic conditions. Analytical-grade sodium hydroxide (supplied by Nanjing Chemical Reagents Co., Ltd., Nanjing, China) was prepared as an aqueous solution at pH 13 to simulate the alkaline effect, evaluating durability under strongly alkaline conditions. Analytical-grade sodium sulfate (supplied by Shanghai Aladdin Biochemical Technology Co., Ltd., Shanghai, China) was prepared as an aqueous solution at pH 7 to simulate the corrosive effect of a neutral saline environment, evaluating the material’s durability under saline conditions.

### 2.2. Test Plan

Based on extensive preliminary experiments, this study determined that the mass ratio of SGPC (SR:GGBS:PG:OPC = 8.0:64.8:7.2:20.0) accounts for 14% of the soil mass [21,22]. The water-to-binder (W/B) ratio was set at 0.49. When the SGPC ratio is 8.0:64.8:7.2:20.0, the hydration reaction proceeds fully, providing a reliable guarantee for the strength development of the stabilized soil. When the SGPC content in the soil is less than 14%, the system lacks sufficient cementitious products (e.g., C-S-H gel and AFt) generated by hydration and pozzolanic reactions, resulting in weak interparticle bonding and low matrix strength; when the SGPC content exceeds 14%, the increase in mechanical strength is limited, while the risk of drying shrinkage cracking increases significantly. As for the W/B ratio, when it is below 0.49, the stabilized soil exhibits poor workability, hindering effective placement and compaction; when it exceeds 0.49, the mix becomes overly fluid, leading to segregation and bleeding. At a W/B ratio of 0.49, the soil shows good workability and favorable conditions for further strength development. Since the natural moisture content of the undisturbed soil was measured to be 43.34%, the W/B ratio of 0.49 includes both the moisture in the soil and the mixing water.

Preliminary screening tests on mechanical properties and erosion resistance indicated that the durability of the solidified soil is optimal at a 15% RCA content; higher RCA contents dilute the cementitious components, increasing interfacial defects and reducing erosion resistance. The core objective of this study is to elucidate the microscopic degradation mechanisms of RCA-modified SGPC-stabilized soil under multi-chemical environments (e.g., acid, alkali, and sulfate). To simplify variables, avoid multivariate coupling, and enable an accurate comparison of durability and microstructural differences between systems with and without RCA, the long-term erosion test was conducted using only the optimal 15% RCA content as the modified group, while the control group contained no RCA; all other admixture proportions were kept identical between the two groups.

The 15% RCA is the external admixture ratio relative to the mass of the original soil; it does not replace any components of the soil or the cementitious material. The cementitious material, SGPC, accounts for 14% of the soil mass, and RCA is added directly to the system as an external admixture, while the total mass of the soil remains unchanged.

Additionally, aqueous solutions of pH 2, 13, and 7 were prepared using analytical-grade hydrochloric acid, sodium hydroxide, and sodium sulfate, respectively, to simulate acidic, alkaline, and neutral saline environments.

### 2.3. Sample Preparation

Following the mix proportions specified above, the raw materials—SR, GGBS, PG, OPC, and RCA—were accurately weighed and poured separately into a JJ-5 planetary mortar mixer (Wuxi Jianyi Laboratory Equipment Co., Ltd., Wuxi, China) for blending. First, dry mixing was performed at a low speed (100–150 r/min) to ensure thorough blending and prevent localized agglomeration. After 30 s, water was slowly added along the inner wall of the mixer. Mixing was continued at a low speed of 100–150 r/min for 2 min, after which the speed was increased to 300–350 r/min and maintained at high speed for another 2 min, ensuring that the slurry was uniform and stable, with no bleeding or segregation.

The prepared slurry was then poured in two batches into standard cylindrical molds (inner diameter 3.91 cm, height 8.00 cm) that had been pre-coated with white petroleum jelly. The filled molds were placed on a vibrating table and compacted for 30 s. The tops of the molds were covered with plastic wrap and allowed to stand at room temperature for 24 h before demolding. After demolding, the appearance of the specimens was carefully inspected; any non-conforming specimens with chipped edges, broken corners, or surface defects were discarded. After the qualified test specimens were transferred to a standard curing chamber (20 ± 2 °C, relative humidity ≥ 95%) and cured for 28 days, the test group was immediately transferred to the corresponding erosion solution for immersion. The erosion periods were 28 days, 90 days, and 120 days, respectively. The control group continued standard curing for 28 days, 90 days, and 120 days, after which weight measurements and mechanical property tests were conducted.

Additionally, specimens cured to the target ages were immersed in anhydrous ethanol for 24 h, then dried in a constant-temperature oven at 60 °C until a constant mass was achieved. Finally, the dried specimens were ground into fine powder using an agate mortar for microstructural analysis.

### 2.4. Test Methods

Flow-cured soil may be subjected to erosion by acids, alkalis, and sulfates in groundwater, industrial wastewater, or chemical environments. Chemical corrosion can damage the cementitious structure, increase porosity, and reduce strength, thereby affecting the material’s long-term durability. Therefore, acid, alkali, and sulfate corrosion tests can quantitatively evaluate the material’s durability performance under complex chemical environments.

For each group, three test specimens and three control specimens were prepared. The specimens were cured in a standard curing chamber until the designated age, while the control group continued standard curing. The test specimens were placed in separate containers containing acidic, alkaline, and saline solutions, respectively, with the liquid level maintained at 20 mm above the top of the specimens, and stored at a constant temperature of 20 ± 2 °C. A sodium sulfate solution with a molarity of 0.35 mol/L was prepared. The pH of the solution was adjusted to 7 using dilute sulfuric acid, and 4.5 L of the solution was added to each set of corrosion test vessels. Every 7 d, the entire corrosion solution was replaced with fresh solution. During replacement, the solution’s pH was measured in real time and adjusted to the initial pH value; pH fluctuations were recorded throughout the process to ensure the stability of ion concentrations in the corrosive medium. The corrosion durations were 28, 90, and 120 d, corresponding to simulated short-term, medium-term, and long-term groundwater chemical corrosion conditions. After corrosion was complete, moisture was wiped from the specimen surfaces, and the mass and unconfined compressive strength of the specimens were measured.

The formulas for calculating the mass loss rate and strength loss rate are as follows:

(1) Mass loss rate
  (1) $$E_{n}=\frac{e_{0}-e_{n}}{e_{0}}\times 100\%$$

In the equation, $E_{n}$—mass loss rate (%); $e_{0}$—initial mass of the test specimen (g); $e_{n}$—mass of the test specimen after erosion (g).

(2) Strength loss rate
  (2) $$H_{n}=\frac{h_{0}-h_{n}}{h_{0}}\times 100\%$$

In the equation, $H_{n}$—strength loss rate (%); $h_{0}$—unconfined compressive strength of the control group (kPa); $h_{n}$—unconfined compressive strength of the specimen after erosion (kPa).

To determine the phase composition of hydration products, X-ray diffraction (XRD) was conducted using a TD-3500 diffractometer (Dandong Tongda Technology Co., Ltd., Dandong, China). The test conditions were as follows: voltage of 30 kV, current of 20 mA, scanning angle range of 5–80°, scanning speed of 0.2°/s, and step size of 0.02°. Microscopic morphological analysis was carried out using a COXEM30 scanning electron microscope (SEM) equipped with an energy-dispersive X-ray spectrometer (EDS) (Beijing Bruker Technology Co., Ltd., Beijing, China). Prior to SEM observation, the samples were coated with a uniform gold layer using an ion sputter coater for 2 min to enhance their conductivity. During testing, the accelerating voltage was set to 15 kV, and the working distance was maintained at 10 mm. For each set of specimens, three distinct block-shaped observation areas in the middle section were selected for morphological observation, with the selection criteria being to avoid areas with human-induced damage from specimen preparation.

In the full-immersion erosion tests used in this study, after the specimens were removed, only the moisture on their outer surfaces was wiped off before weighing; three specimens were measured for each test group, and the arithmetic mean was calculated. This conventional testing method cannot quantitatively distinguish between mass increases caused by hydration, water absorption into the pores, and the accumulation of salt crystals within the pores. The measured mass change is the combined result of these multiple coupled effects, which constitutes an inherent limitation of this testing method. Therefore, this study did not rely solely on mass change data to assess the extent of hydration reactions. The mass change indicator was used solely for relative comparative analysis among different groups; the quantitative assessment of the evolution of hydration products relied primarily on TG–DTG thermogravimetric analysis, combined with XRD and SEM microscopic characterization, as well as a comprehensive analysis of the strength loss rate, to avoid interference caused by the coupling of multiple factors associated with macroscopic mass indicators.

## 3. Results and Discussion

### 3.1. Analysis of Macroscopic Properties Under Different Chemical Corrosion Conditions

Table 3 shows the changes in the strength of the specimens under different chemical corrosion environments. Table 4 and Table 5 present the strength loss rates and mass loss rates of the specimens under different chemical erosion environments. As shown in the tables, the evolution patterns of both strength loss and mass loss of the stabilised soil vary with the type of erosive medium and the duration of exposure. In a strong acid erosion environment, H^+^ damages the C-S-H gel and AFt hydration products [23]. After 120 d, the strength loss rate and mass loss rate of the SGPC control group reached 19.86% and 4.33%, respectively, whereas those of the SGPC-RCA group (with 15% RCA added) fell to 9.02% and 1.46%, respectively. RCA optimises the pore structure through a physical skeleton-filling effect and reduces ion transport capacity, thereby effectively mitigating acid-induced degradation; however, it cannot eliminate the chemical dissolution of hydration products, and the acidic environment remains the most unfavourable service condition for this system [24]. Under strong alkali erosion, secondary hydration occurs in the early-stage OH^−^-activated system and on the surface of the old mortar in the RCA group [25]. Both groups of specimens exhibited increases in strength and mass; as the curing age increased, only slight surface damage was observed. The mass loss rate of the 120 d SGPC-RCA specimens was only 0.33%, far lower than the 1.46% of the SGPC group. Although the increase in the number of aggregate-paste interfaces results in greater strength gains, the RCA skeleton effect ensures the overall structural integrity of the specimens [26]. Sulphate erosion exhibited characteristics of early-stage pore filling followed by late-stage expansion damage; the continuous reaction of SO_4_^2−^ produced excess AFt, leading to expansion and cracking [27]. In the SGPC control group, the strength loss rate and mass loss rate at 120 d were 5.65% and 2.98%, respectively, whereas in the SGPC-RCA group these values decreased to 2.02% and 1.66%. Guo et al. [28] demonstrated through uniaxial compression tests that recycled coarse aggregate can form a rigid skeletal structure within the cement matrix, effectively bearing and distributing external loads. A comprehensive comparison of the macroscopic indicators at 120 d reveals that the erosion resistance of this solidified soil based on multi-source solid waste follows the order: strongly alkaline environment > sulphate environment > strongly acidic environment. The incorporation of 15% RCA enhances the macroscopic durability of the solidified soil against chemical erosion by acids, alkalis and sulphates through the combined effects of pore regulation, skeletal support, and the induction of secondary hydration.

### 3.2. Phase Analysis of Hydration Products Under Different Erosion Conditions

**Figure 1:**
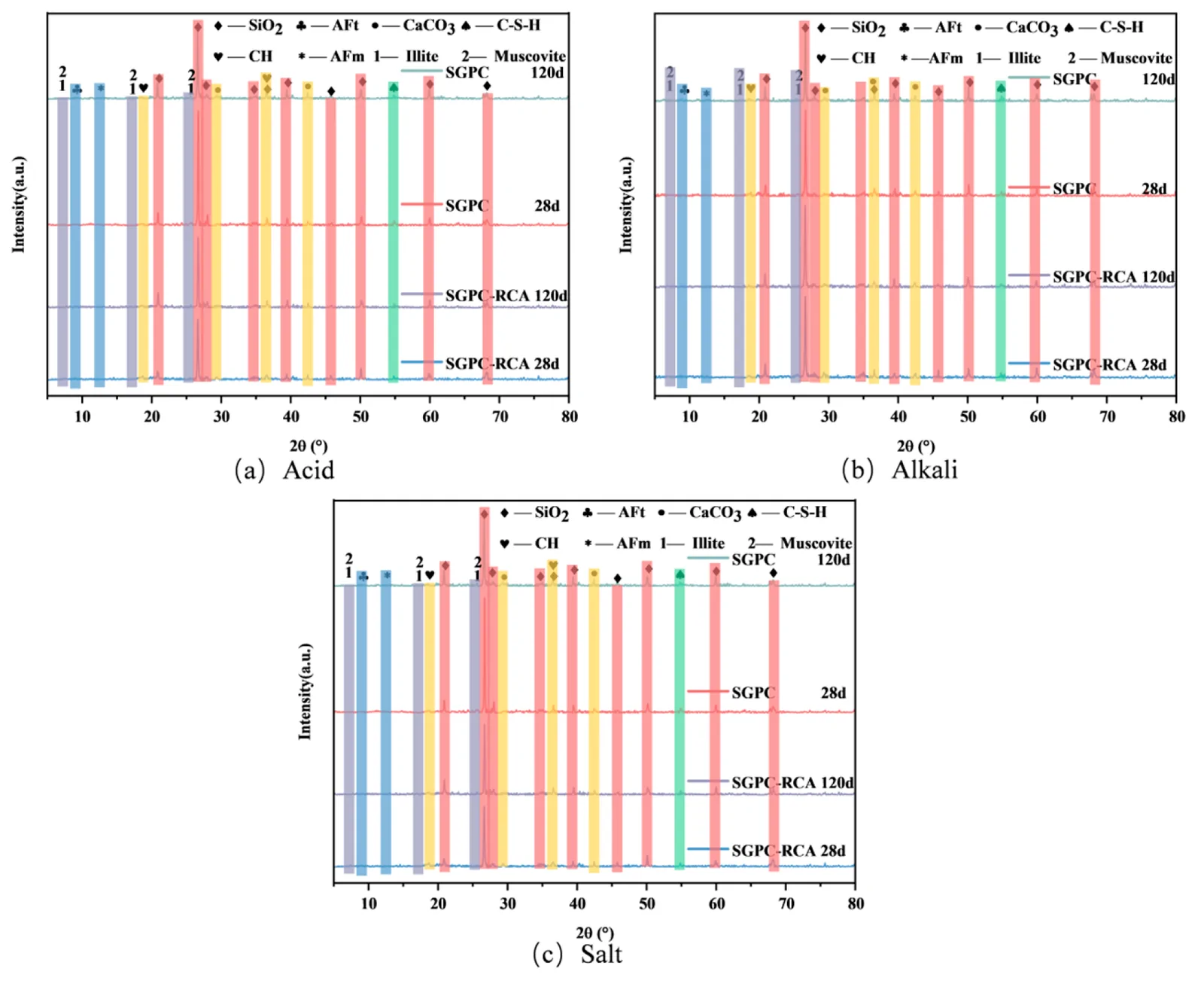
XRD patterns under different erosion conditions.

Figure 1 shows the XRD patterns of the specimens under different chemical erosion conditions. As shown in the patterns, the main phases in all specimens consist of SiO_2_, AFt, CaCO_3_ and C-S-H gel. While the types of phases remain unchanged, the relative diffraction peak intensities of each phase exhibited distinct variation trends, depending on the type of erosion medium, the duration of erosion, and whether RCA was added. Under acid erosion conditions, as the erosion age increased from 28 d to 120 d, the relative diffraction peak intensities of AFt and C-S-H in the SGPC control group (without RCA addition) exhibited a decreasing trend, indicating that H^+^ in the acidic environment dissolved and decomposed the hydrated gel products [29]. At the same erosion age, the relative diffraction peak intensities of AFt and C-S-H in the SGPC-RCA specimens tended to be higher than those in the SGPC control group, suggesting that the incorporation of RCA inhibited the penetration of erosive ions into the matrix, effectively protecting the hydrated gel products and mitigating the dissolution and degradation of the hydrated phase by the acidic medium [30].

In the strongly alkaline erosion system, both the SGPC and SGPC-RCA specimen groups maintained relatively high relative diffraction peak intensities of AFt and C-S-H over the age range of 28 to 120 d, with no significant dissolution or degradation of the hydration products observed. Compared with the SGPC control group, the characteristic C-S-H diffraction peaks of the SGPC-RCA specimens exhibited relatively higher intensities, confirming that, under strongly alkaline conditions, the old mortar on the RCA surface could be activated and undergo secondary hydration reactions, resulting in the formation of more C-S-H gel within the system, which corresponded to the observed increase in macroscopic strength.

In the sulfate-corrosive environment, the relative diffraction peak intensity of AFt in the 120 d specimens of the SGPC control group exhibited an increasing trend compared with that at 28 d, indicating the extensive formation of expansive AFt within the system. In a sulfate corrosive environment, on the contrary, through the physical support provided by its rigid framework, it inhibited the propagation of microcracks, thereby mitigating the expansion damage caused by excessive AFt crystallization. This explains, at the phase level, the underlying reason for the improved resistance to sulfate corrosion in RCA-modified solidified soil.

Overall, the introduction of RCA did not alter the phase composition of the system; rather, it regulated the chemical erosion resistance of solid waste-based solidified soil primarily by slowing down the consumption of hydration products, promoting secondary hydration to form a cementitious phase.

### 3.3. Microscopic Topography Analysis Under Different Erosion Conditions

**Figure 2:**
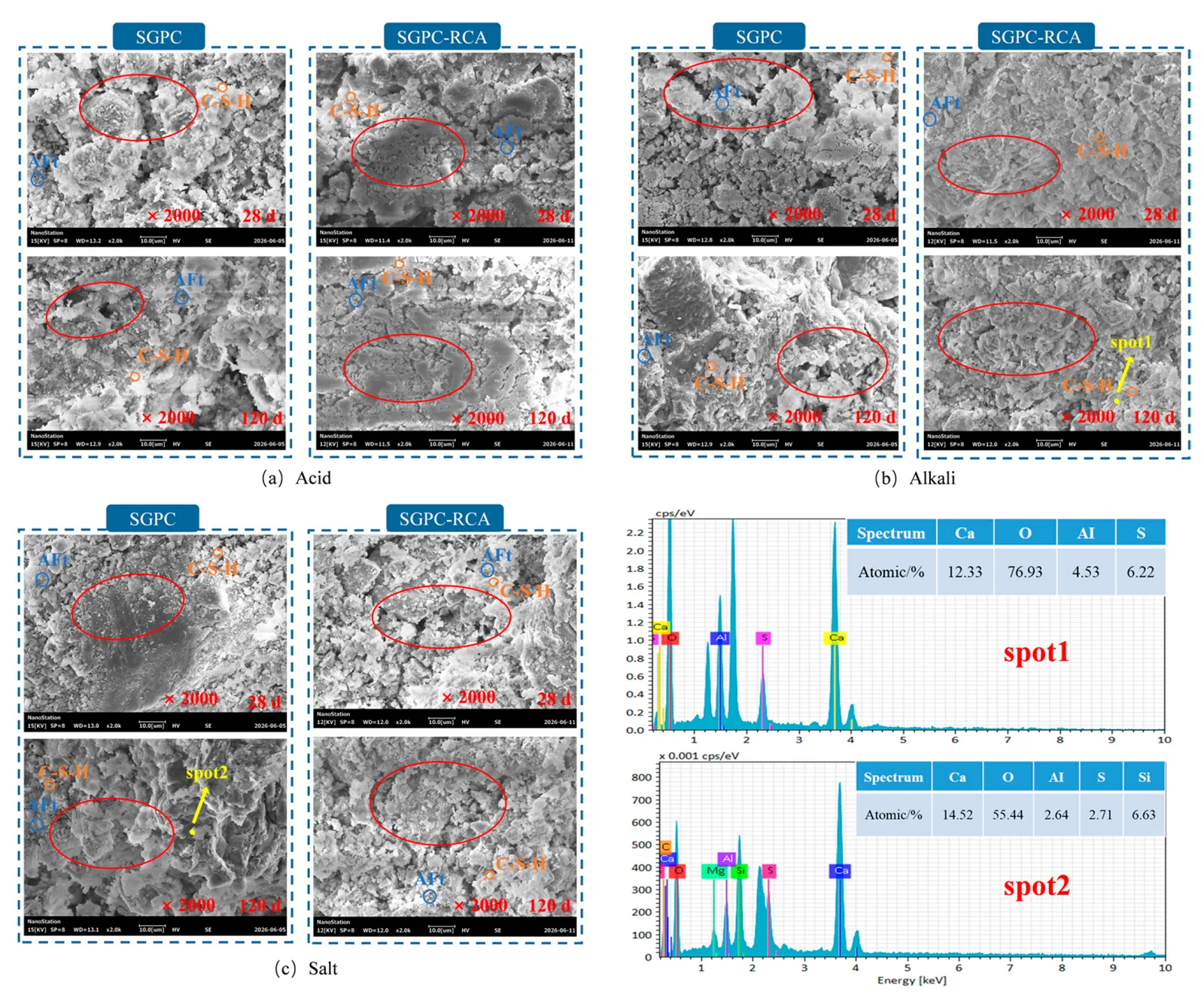
SEM images showing different erosion processes.

Figure 2 shows the SEM images of the specimens. The scanning electron microscopy results clearly reveal the microstructural characteristics of the SGPC and SGPC RCA specimens, as well as the morphological evolution of their hydration products under different erosion media and at different erosion durations.

Under strong acid erosion, after 28 d of exposure, both groups of specimens still exhibited a certain degree of continuous gel structure in their matrices. However, by 120 d, the matrix of the SGPC control group had been significantly damaged by H^+^ dissolution: the gel network became fragmented and loose, with numerous voids visible internally, indicating that the structural integrity of the hydration products had been compromised. In contrast, the SGPC RCA specimen matrix showed better overall continuity, with fewer voids; the hydration gels interlocked to form a relatively dense matrix, and only slight dissolution traces were observed in localized areas. This confirms that RCA can refine the pore structure and delay the erosive damage to the matrix microstructure caused by acidic media, although it cannot completely inhibit the microstructural degradation resulting from acid erosion. Under strong alkaline corrosion, extensive flocculent and flaky C-S-H gel formation was observed in the 28 d SGPC RCA specimens, which served to fill the interfaces between aggregates and paste as well as internal voids [31]. At 120 d, the SGPC RCA matrix remained generally dense; although the matrix of the SGPC control group was also relatively dense, it contained more internal voids than that of the SGPC RCA group. These microstructural observations further corroborate that, under strongly alkaline conditions, the old mortar on the RCA surface underwent secondary hydration, and the newly formed cementitious phase achieved a self-compacting effect within the matrix [32]. Under sulfate corrosion, as the exposure age increased, a large accumulation of needle- and rod-shaped AFt expansion crystals was observed within the SGPC control group’s matrix at 120 d, accompanied by significant microstructural damage. In the SGPC RCA specimens, the matrix integrity was better preserved, and microcracks were effectively suppressed [33]. This indicates that the rigid RCA framework can share and disperse the internal expansion stresses generated by sulfate corrosion, thereby mitigating damage caused by expansion cracking.

Overall, the SEM microscopic observations corroborate the aforementioned macroscopic strength and mass loss trends, as well as the XRD phase evolution patterns, and they elucidate at the microstructural level the mechanism by which RCA enhances the chemical corrosion resistance of solidified soil made from multi-source solid waste.

### 3.4. Quantitative Analysis of Hydration Products Under Different Erosion Conditions

**Figure 3:**
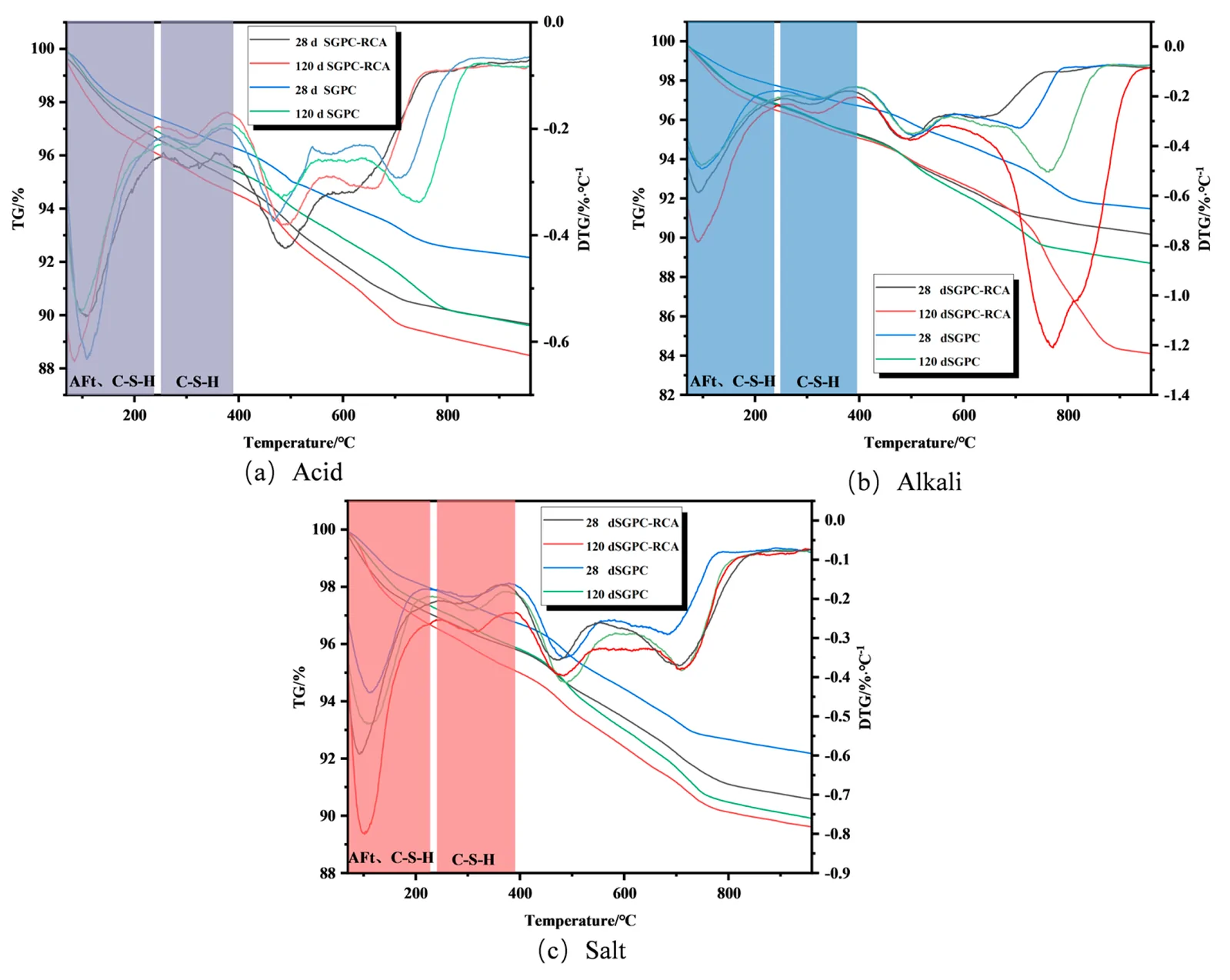
TG-DTG curves under different erosion conditions. The smooth curve corresponds to the TG curve on the left TG axis. The wavy curve corresponds to the DTG curve on the right DTG axis.

Figure 3 shows the TG-DTG curves of the samples. The figure highlights two typical regions of mass loss: the 50–200 °C range corresponds to the dehydration of free water, AFt and C-S-H [34], and mass loss in the 200–400 °C range is primarily due to the dehydroxylation of C-S-H gel, but also includes contributions from the decomposition of AFt, AFm, calcium aluminate hydrate, and other aluminate hydrates [35]. In this study, the total mass loss rate in this characteristic temperature range was used to semi-quantitatively characterize the trend in the total amount of hydration products. Table 6 presents the mass loss rates for the various stages of the different samples. Under acid erosion conditions, as the erosion age increases from 28 d to 120 d, the total mass loss in the characteristic temperature range of the hydration products for the SGPC control group decreases from 1.99% to 1.84%, whereas that for the SGPC-RCA specimens decreases from 2.38% to 2.25%. The SGPC control group exhibits greater mass loss variations within the temperature ranges corresponding to AFt and C-S-H, indicating that the acidic environment causes the dissolution and decomposition of a large amount of AFt and C-S-H hydration and cementitious products [36]. Under the same ageing conditions, the mass loss of the SGPC-RCA specimens within this temperature range is significantly higher than that of the SGPC control group, indicating a higher initial content of hydration products in the matrix; simultaneously, the rate of mass loss decline at 120 d is smaller, suggesting that the introduction of RCA effectively retains the hydration products within the system and inhibits the degradation of the cementitious phase by the acidic medium. Under strong alkali erosion conditions, the mass loss in the characteristic range of hydration products for the SGPC specimens varies from 3.17% to 3.03%, whilst that for the SGPC-RCA specimens ranges from 3.23% to 3.22%. Both groups of specimens exhibit distinct mass loss peaks in the dehydration range of hydration products. Compared with the SGPC specimens, the SGPC-RCA specimens show greater mass loss in the AFt and C-S-H dehydration temperature ranges, and this mass loss shows almost no decrease with ageing, indicating that RCA triggers secondary hydration in a strong alkali environment, leading to an increase in the formation of cementitious products such as C-S-H within the system. As the erosion period extends to 120 d, the SGPC-RCA specimens still maintain a high retention of hydration products, demonstrating their excellent resistance to alkali erosion. Under sulphate erosion conditions, between 28 d and 120 d, the mass loss of the SGPC specimens in the characteristic hydration product range decreases from 2.99% to 2.52%, whilst the mass loss of the SGPC-RCA specimens in the same range decreases from 3.08% to 2.97%. The SGPC control group at 120 d shows a significant reduction in mass loss within the AFt characteristic temperature range, which indirectly corresponds to the formation of a large amount of expansive AFt within the system that consumes the original cementitious phase. In a sulfate corrosive environment, through the physical support provided by its rigid framework, it inhibited the propagation of microcracks, thereby mitigating the expansion damage caused by excessive AFt crystallization. The SGPC-RCA group still maintained a high content of hydration products at 120 days, and its strength loss rate was significantly lower, indicating that RCA enhances resistance to sulfate corrosion through a structural reinforcement mechanism rather than by inhibiting the formation of AFt [37]. The quantitative results of the thermogravimetric analysis are corroborated by macroscopic properties, XRD phase analysis and SEM microstructural observations, further elucidating the intrinsic mechanism by which RCA enhances the resistance of stabilised soil to multi-chemical attack from the perspective of hydration product content.

### 3.5. Mechanism Analysis

Among the three types of chemical corrosion conditions—acidic, alkaline, and saline—this multi-source solid waste-based fluidized solidified soil exhibits the best adaptability and durability in alkaline environments, followed by neutral sulfate environments, and shows the poorest corrosion resistance under strongly acidic conditions. Strongly alkaline environments can trigger secondary hydration reactions in the solidified system; the newly formed hydration products fill internal pores, causing an initial increase in the mass of the test specimens. Under long-term erosion, only minor surface dissolution occurs, resulting in a relatively low overall degree of degradation. In the SGPC-RCA system, the old mortar adhering to the RCA surface can be further activated by the alkaline environment, promoting the continuous progression of hydration reactions and further enhancing matrix density; mass loss over the entire exposure period is significantly lower than in the other two erosion environments. The increase in the number of aggregate-paste interfaces resulted in a slight increase in the strength of SGPC-RCA compared to the control group, and the skeletal support provided by the RCA ensured the overall structural integrity of the specimens. XRD spectra show that the phase composition of the hydration products is stable, with no significant attenuation of characteristic peaks; SEM observations reveal a continuous and dense cementitious network, with minimal formation of interfacial defects and microcracks. Both macroscopic performance and microscopic characterization confirm that this solidification system exhibits optimal chemical and structural stability in a strongly alkaline environment, rendering it an effective approach for improving the durability of solidification materials based on multi-source solid waste.

This schematic diagram systematically illustrates the microstructural evolution and degradation mechanisms of RCA-modified solid waste-based solidification materials under three typical chemical erosion environments (hydrochloric acid, sodium sulfate, and sodium hydroxide). The overall durability of these materials follows the order alkaline environment > sulfate environment > acidic environment, with dissolution and decomposition mechanisms of C-S-H gel and AFt under H^+^ erosion, and the pore-filling effect of RCA; secondary hydration reactions of residual mortar on the RCA surface and the formation of large amounts of hydration products under OH^−^ erosion; and expansion, crystallization, and microcrack propagation of AFt under SO_4_^2−^ erosion. Under HCl corrosion, the rapid penetration of H^+^ triggers the destruction of C-S-H gel and AFt, leading to severe disintegration and collapse of the cementitious matrix [38]. Although RCA can slightly delay ion diffusion through physical filling, it cannot prevent the chemical dissolution of hydration products, resulting in the poorest long-term durability. The study by Zou et al. [39] further demonstrates that, during sulfate corrosion, the rigid support provided by aggregates can inhibit the propagation of microcracks caused by the crystallization of expansion products (AFt). The “rigid aggregate—stress dissipation—crack inhibition” mechanism revealed in the aforementioned literature provides a reliable theoretical framework for this study.

**Figure 4:**
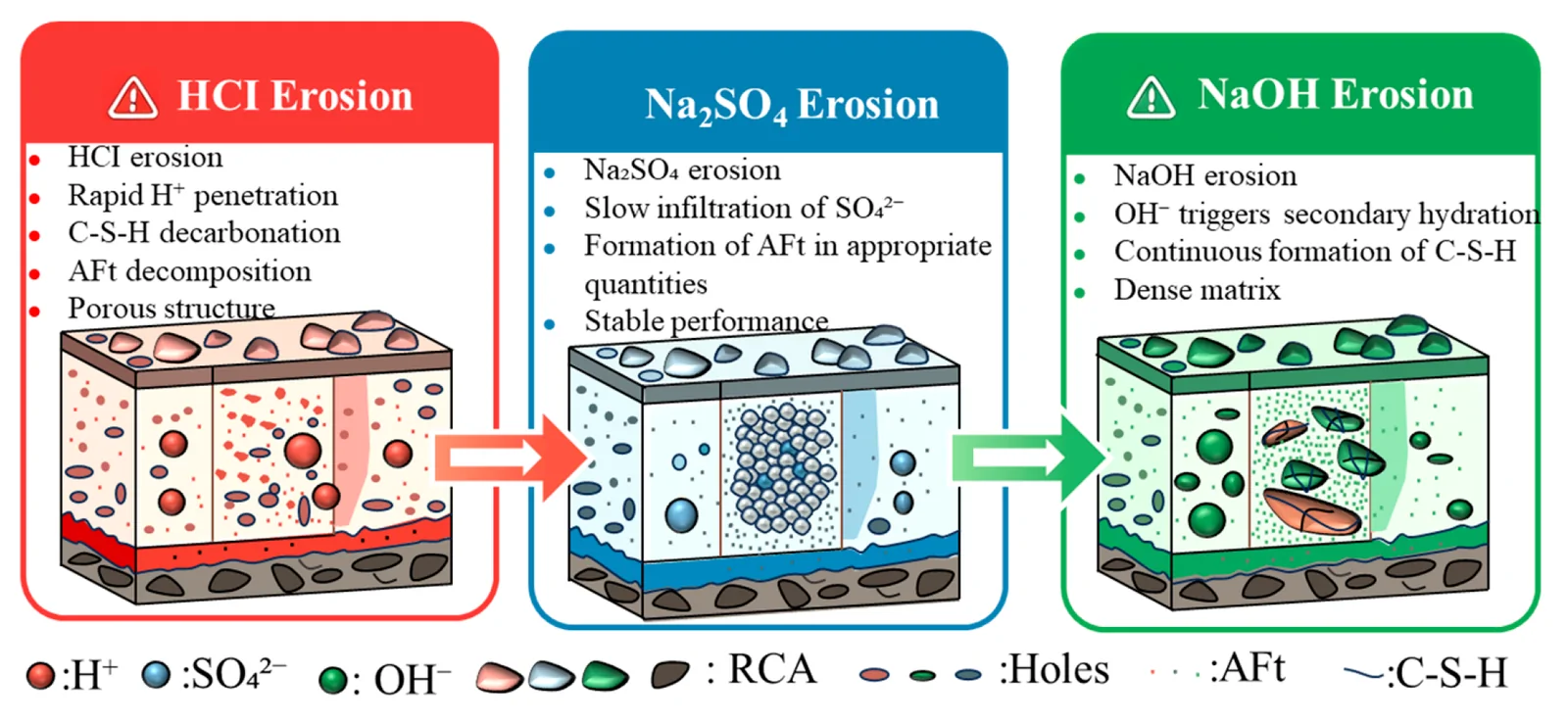
Schematic Diagrams of Mechanisms Under Different Erosion Conditions.

Figure 4 shows a schematic diagram of the mechanisms at work under different erosion conditions. The rigid RCA framework effectively disperses internal expansion stress and inhibits crack propagation, thereby significantly mitigating sulfate corrosive damage and demonstrating moderate durability performance. Under NaOH alkali attack, high concentrations of OH^−^ continuously activate the secondary hydration reaction of residual mortar on the RCA surface; a large amount of newly formed C-S-H gel fills the interconnected pores and interfacial transition zones, thereby achieving self-densification of the matrix [40]. The hydration products (C-S-H and AFt) maintain excellent chemical stability even under long-term alkaline conditions, thereby ensuring optimal structural integrity and durability. This schematic diagram illustrates the differential roles of RCA under the three chemical corrosion conditions from three perspectives—mass transfer, phase evolution, and structural damage—providing a theoretical basis for the engineering application of solid waste-based solidification materials in complex contaminated sites. It was noted that SR contains high levels of chloride ions, posing a potential environmental risk of chloride leaching when used in soil stabilization projects; this study focused solely on mechanical durability under chemical erosion and did not evaluate leaching behavior.

## 4. Conclusions

This study prepared a composite cementitious binder by blending SR, GGBS, PG, and OPC, and incorporated RCA-modified fluidized solidified soil for performance testing and microscopic analysis under various chemical erosion conditions and exposure durations. The main conclusions are as follows:

(1) Under the three types of chemical erosion environments, the SGPC-RCA group (incorporating 15% RCA) exhibits significantly lower mass and strength loss rates after 120 d of exposure compared to the control group, indicating that the introduction of RCA effectively enhances the long-term service stability of the solidified material in complex chemical environments.
(2) Macroscopic-durability indicators combined with XRD, SEM and TG-DTG results indicate that RCA addition produces a denser matrix state. It can mitigate the degradation originating from dissolution and expansion-driven cracking of hydration products under chemical attack.
(3) Under alkaline corrosion environments, RCA-modified specimens show favourable durability performance. Existing test results imply that residual old-mortar fractions on RCA may participate in secondary-hydration reactions, while the rigid-aggregate skeleton helps maintain matrix integrity.

## Figures and Tables

**Table 1:** Basic physical properties of untreated soil.

Physical Indicator	Water Content (%)	Liquid Limit (%)	Plastic Limit (%)	Plasticity Index	Specific Gravity	Maximum Dry Density (g/cm^3^)	pH
Value	43.34	43.27	22.39	20.83	2.67	1.79	6.44

**Table 2:** Main chemical components of the hardener.

Raw Materials	SiO_2_	CaO	Al_2_O_3_	Fe_2_O_3_	MgO	SO_3_	P_2_O_5_	Cl^−^	Loss on Ignition LOI	Other
GGBS	32.80	39.50	14.10	1.60	7.50	0.50	—	—	2.10	1.90
PG	3.10	37.70	0.35	0.30	0.14	41.50	2.31	—	13.20	1.40
SR	10.30	42.20	3.20	0.80	5.08	6.20	1.02	27.30	3.10	0.80
OPC	21.50	58.40	7.50	1.80	2.00	5.10	—	—	3.20	0.50

**Table 3:** Changes in strength under different chemical erosion conditions.

Erosion Environment	Group	Age (d)	Control Group (MPa)	Experimental Group (MPa)			Standard Deviation
Acid	SGPC	28	2.22	2.02	2.01	1.99	0.25239
		90	2.46	2.05	2.04	2.03	0.24576
		120	2.87	2.31	2.29	2.26	0.24576
	SGPC-RCA	28	2.69	2.60	2.58	2.56	0.19519
		90	2.94	2.73	2.70	2.71	0.34117
		120	3.28	2.99	2.98	2.97	0.15133
Alkali	SGPC	28	2.46	2.60	2.59	2.62	0.34589
		90	2.59	2.77	2.79	2.77	0.14526
		120	2.60	3.09	3.11	3.09	0.34589
	SGPC-RCA	28	2.96	3.32	3.35	3.33	0.35987
		90	3.28	3.83	3.85	3.83	0.16965
		120	3.54	4.18	4.22	4.20	0.51023
Sulfate	SGPC	28	2.34	2.36	2.34	2.37	0.18234
		90	2.49	2.41	2.38	2.40	0.32986
		120	2.79	2.35	2.34	2.36	0.25032
	SGPC-RCA	28	2.64	2.77	2.79	2.78	0.14107
		90	2.85	2.83	2.81	2.80	0.28431
		120	3.32	3.26	3.24	3.24	0.19925

**Table 4:** Strength loss rates (%) under different chemical erosion conditions.

Erosion Condition	Sample	Age/d		
		28 d	90 d	120 d
Acid	SGPC	9.27	16.82	19.86
	SGPC-RCA	3.64	7.66	9.02
Alkali	SGPC	−5.24	−7.23	−9.66
	SGPC-RCA	−12.63	−16.89	−18.56
Sulfate	SGPC	−1.12	3.36	5.65
	SGPC-RCA	−5.66	1.13	2.02

Note: ‘−’ indicates that the unconfined compressive strength of the eroded specimen was higher than that of the control group cured under standard conditions.

**Table 5:** Mass loss rates (%) under different chemical erosion conditions.

Erosion Condition	Sample	Age/d		
		28 d	90 d	120 d
Acid	SGPC	2.16	3.28	4.33
	SGPC-RCA	0.56	1.06	1.46
Alkali	SGPC	−0.81	0.99	1.46
	SGPC-RCA	−2.39	0.18	0.33
Sulfate	SGPC	−0.36	1.81	2.98
	SGPC-RCA	−1.15	1.02	1.66

Note: ‘−’ indicates that the mass of the sample after erosion was higher than that of the standard-cured control group.

**Table 6:** Weight loss rates (%) under different erosion conditions.

Erosion Condition	Sample	28 d		120 d	
		AFt, C-S-H Weight Loss Rate	C-S-H Weight Loss Rate	AFt, C-S-H Weight Loss Rate	C-S-H Weight Loss Rate
Acid	SGPC	1.99	0.83	1.84	0.98
	SGPC-RCA	2.38	1.09	2.25	0.90
Alkali	SGPC	3.17	1.28	3.03	1.30
	SGPC-RCA	3.23	1.26	3.22	1.14
Sulfate	SGPC	2.99	1.19	2.52	0.94
	SGPC-RCA	3.08	1.28	2.97	1.31

## Data Availability

The original contributions presented in this study are included in the article. Further inquiries can be directed to the corresponding authors.
